# Spatio-Temporal Backpropagation for Training High-Performance Spiking Neural Networks

**DOI:** 10.3389/fnins.2018.00331

**Published:** 2018-05-23

**Authors:** Yujie Wu, Lei Deng, Guoqi Li, Jun Zhu, Luping Shi

**Affiliations:** ^1^Department of Precision Instrument, Center for Brain-Inspired Computing Research, Beijing Innovation Center for Future Chip, Tsinghua University, Beijing, China; ^2^Department of Electrical and Computer Engineering, University of California, Santa Barbara, Santa Barbara, CA, United States; ^3^State Key Lab of Intelligence Technology and System, Tsinghua National Lab for Information Science and Technology, Tsinghua University, Beijing, China

**Keywords:** spiking neural network (SNN), spatio-temporal recognition, leaky integrate-and-fire neuron, MNIST-DVS, MNIST, backpropagation, convolutional neural networks (CNN)

## Abstract

Spiking neural networks (SNNs) are promising in ascertaining brain-like behaviors since spikes are capable of encoding spatio-temporal information. Recent schemes, e.g., pre-training from artificial neural networks (ANNs) or direct training based on backpropagation (BP), make the high-performance supervised training of SNNs possible. However, these methods primarily fasten more attention on its spatial domain information, and the dynamics in temporal domain are attached less significance. Consequently, this might lead to the performance bottleneck, and scores of training techniques shall be additionally required. Another underlying problem is that the spike activity is naturally non-differentiable, raising more difficulties in supervised training of SNNs. In this paper, we propose a spatio-temporal backpropagation (STBP) algorithm for training high-performance SNNs. In order to solve the non-differentiable problem of SNNs, an approximated derivative for spike activity is proposed, being appropriate for gradient descent training. The STBP algorithm combines the layer-by-layer spatial domain (SD) and the timing-dependent temporal domain (TD), and does not require any additional complicated skill. We evaluate this method through adopting both the fully connected and convolutional architecture on the static MNIST dataset, a custom object detection dataset, and the dynamic N-MNIST dataset. Results bespeak that our approach achieves the best accuracy compared with existing state-of-the-art algorithms on spiking networks. This work provides a new perspective to investigate the high-performance SNNs for future brain-like computing paradigm with rich spatio-temporal dynamics.

## 1. Introduction

Spiking neural network encodes information in virtue of the spike signals and shall be promising to effectuate more complicated cognitive functions in a way most approaching to the processing paradigm of brain cortex (Allen et al., 2009; Zhang et al., 2013; Kasabov and Capecci, 2015). SNNs are advantageous primarily due to the following two aspects: (1) more spatio-temporal information is encoded with spike pattern flows through SNNs, whereas most DNNs lack timing dynamics, especially the extensively-adopted feedforward DNNs; (2) more benefits can be achieved the hardware in virtue of the event-driven paradigm of SNNs, which has been leveraged by numerous neuromorphic platforms (Benjamin et al., 2014; Furber et al., 2014; Merolla et al., 2014; Esser et al., 2016; Hwu et al., 2016; Zhang et al., 2016).

Yet the SNNs training still remains challenging because of the quite complicated dynamics and non-differentiable nature of the spike activity. In a nutshell, the existing training methods for SNNs fall into three types: (1) unsupervised learning; (2) indirect supervised learning; (3) direct supervised learning. The first one is originated from the weight modification of biological synapses, e.g., spike timing dependent plasticity (STDP) (Querlioz et al., 2013; Diehl and Cook, 2015; Kheradpisheh et al., 2016). Stemmed from its primary dependency on the local neuronal activities without global supervisor, effectuating high performance is quite difficult. The second one firstly trains an ANN, and thereupon transforms it into its SNN version with the same network structure where the rate of SNN neurons acts as the analog activity of ANN neurons (Peter et al., 2013; Hunsberger and Eliasmith, 2015; Neil et al., 2016). The last one is the direct supervised learning. Gradient descend, is a very popular optimization method for this learning type (Bohte et al., 2000; Schrauwen and Campenhout, 2004; Mckennoch et al., 2006; Lee et al., 2016). Spikeprop (Bohte et al., 2000; Schrauwen and Campenhout, 2004; Mckennoch et al., 2006) pioneered the gradient descent method to design multi-layer SNNs for supervised learning. It uses the first-spike time to encode input signals and minimizes the difference between the network output and desired signals, the whole process of which is similar to the traditional BP. Lee et al. (2016) treated the membrane potential as differentiable signals to solve the non-differential problems of spikes, and proposed a directly BP algorithm to train deep SNNs. Another efficient directly learning type is based on biological synaptic plasticity mechanism. Ponulak (2005); Ponulak and Kasiski (2010) developed the ReSuMe algorithm which uses STDP-like rule with remote supervision to learn the desired output spike sequence. Gtig and Sompolinsky (2006); Urbanczik and Senn (2009) proposed the tempotron learning rule which embeds information into spatio-temporal spike pattern and modifies synaptic connection by the output spike signals. Besides, some researchers utilized the unsupervised local plasticity mechanisms to abstract hierarchical features, and further modified network by parameters label signals (Mozafari et al., 2017; Tavanaei and Maida, 2017). Many emergent supervised training methods for SNNs have considered the spatial-temporal dynamic of spike-based neuron, but most of them primarily fasten more attention on one side of feature, either the spatial feature or the temporal feature, which in essence does not play out the advantage of SNNs and have to leverage several complicated skills to improve performance, such as error normalization, weight/threshold regularization, specific reset mechanism, etc. (Diehl et al., 2015; Lee et al., 2016; Neil et al., 2016). To this end, it is meaningful to design more general dynamic model and learning algorithm on SNNs.

In this paper, a direct supervised learning method is proposed for SNNs, combining both the spatial domain (SD) and temporal domain (TD) in the training phase. First and foremost, an iterative LIF model with SNNs dynamics is established which is appropriate for gradient descent training. On that basis, both the spatial dimension and temporal dimension are considered during the error backpropagation (BP) to evidently improves the network accuracy. An approximated derivative is introduced to address the non-differentiable issue of the spike activity. We test our SNNs model through adopting both the fully connected and convolutional architecture on the static MNIST dataset and a custom object detection dataset, as well as the dynamic N-MNIST dataset. Our method can make full use of spatio-temporal-domain (STD) information that captures the nature of SNNs, thus avoiding any complicated training skill. Experimental results indicate that the proposed method could achieve the best accuracy on both static and dynamic datasets compared with existing state-of-the-art algorithms. The influence of TD dynamics and different methods for the derivative approximation are analyzed systematically. This work enables to explore the high-performance SNNs for future brain-like computing paradigms with rich STD dynamics.

## 2. Methods and materials

We focus on how to efficiently train SNNs by taking full advantage of the spatio-temporal dynamics. In this section, we propose a learning algorithm that enables us to apply spatio-temporal BP for training spiking neural networks. To this end, subsection 2.1 firstly introduces an iterative leaky integrate-and-fire (LIF) model that are suitable for the error BP algorithm; subsection 2.2 gives the details of the proposed STBP algorithm; subsection 2.3 proposes the derivative approximation to address the non-differentiable issue.

### 2.1. Iterative leaky integrate-and-fire model in spiking neural networks

**Figure 1:**
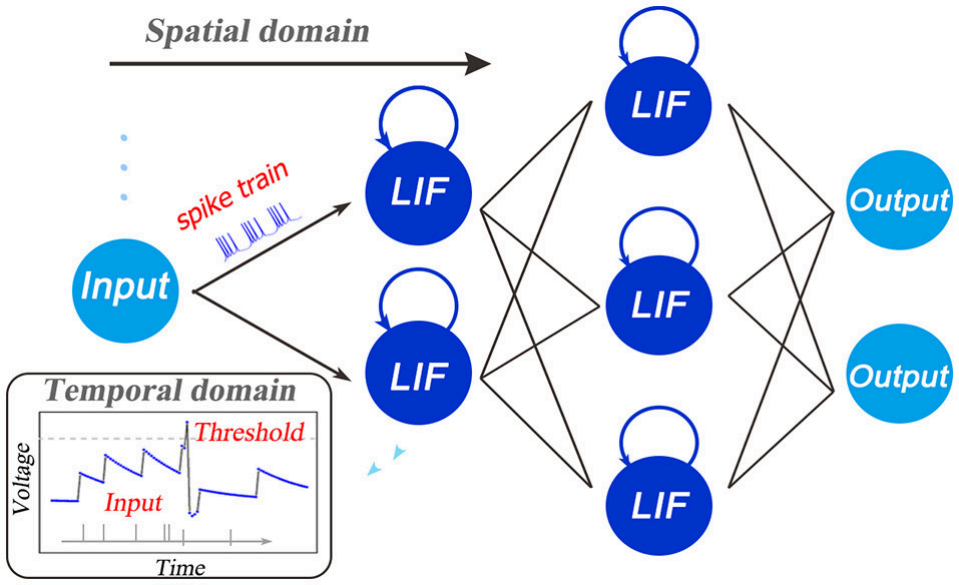
Illustration of the spatio-temporal characteristic of SNNs. In addition to the layer-by-layer spatial dataflow like ANNs, SNNs are famous for the rich temporal dynamics. The existing training algorithms primarily fasten more attention on one side, either the spatial domain such as the supervised ones via backpropagation, or the temporal domain such as the unsupervised ones via timing-based plasticity. This causes the performance bottleneck. Therefore, how to build a framework for training high-performance SNNs by making full use of the STD information forms the major motivation of this work.

It is known that Leaky Integrate-and-Fire (LIF) is the most commonly used model at present to describe the neuronal dynamics in SNNs, and it can be simply governed by:

(1) $$\begin{array}{r} \tau \frac{du\left(t\right)}{dt}=-u\left(t\right)+I\left(t\right) \end{array}$$

where *u*(*t*) is the neuronal membrane potential at time *t*, τ is a time constant and *I*(*t*) denotes the pre-synaptic input which is determined by the pre-neuronal activities or external injections and the synaptic weights. When the membrane potential *u* exceeds a given threshold *V*_*th*_, the neuron fires a spike and resets its potential to *u*_*reset*_. As shown in Figure 1, the forward dataflow of the SNN propagates in the layer-by-layer SD like DNNs, and the self-feedback injection at each neuron node generates non-volatile integration in the TD. In this way, the whole SNNs run with complex STD dynamics and code spatio-temporal information into the spike pattern. The existing training algorithms only consider either the SD such as the supervised ones via BP, or the TD such as the unsupervised ones via timing-based plasticity, which might cause the performance bottleneck. Therefore, how to build a learning model by taking full use of the spatio-temporal domain (STD) forms the main motivation of this work.

However, directly obtaining the analytic solution of LIF model in (1) makes it inconvenient to train SNNs based on BP with discrete dataflow. This is because the whole network presents complex dynamics in continuous TD. To address this issue, firstly we solve the linear differential Equation (1) with the initial condition *u*(*t*)|_*t*=*t*_*i*−1__ = *u*(*t*_*i*−1_), and get the following iterative updating rule:

(2) $$\begin{array}{r} u\left(t\right)=u\left(t_{i-1}\right)e^{\frac{t_{i-1}-t}{\tau }}+\hat{I}\left(t\right) \end{array}$$

where the neuronal potential *u*(*t*) in (1) depends on the previous potential at time *t*_*i*−1_ and the general pre-synaptic input $\hat{I}$(*t*). The membrane potential exponentially decays until the neuron receives new input, and a new update round will start once the neuron fires a spike. That is to say, the neuronal state is co-determined by the spatial accumulations of $\hat{I}$(*t*) and the leaky temporal memory of *u*(*t*_*i*−1_).

As we know, the efficiency of error BP for training DNNs greatly benefits from the iterative representation of gradient descent which yields the chain rule for layer-by-layer error propagation in the SD backward pass. This motivates us to propose an iterative LIF based SNNS in which the iterations occur in both the SD and TD as follows:

(3) $$\begin{array}{rc} x_{i}^{t+1,n} & =\overset{l\left(n-1\right)}{\underset{j=1}{\sum }}w_{ij}^{n}o_{j}^{t+1,n-1} \end{array}$$

(4) $$\begin{array}{rc} u_{i}^{t+1,n} & =u_{i}^{t,n}f\left(o_{i}^{t,n}\right)+x_{i}^{t+1,n}+b_{i}^{n} \end{array}$$

(5) $$\begin{array}{rc} o_{i}^{t+1,n} & =g\left(u_{i}^{t+1,n}\right) \end{array}$$

where

(6) $$\begin{array}{rc} f\left(x\right) & =\tau e^{-\frac{x}{\tau }} \end{array}$$

(7) $$\begin{array}{r} g\left(x\right)=\left\{ \begin{array}{cc} 1,\; & x\geq V_{th}\\ 0,\; & x<V_{th} \end{array}\right. \end{array}$$

In above formulas, the upper index *t* denotes the time step *t*, and *n* and *l*(*n*) denote the *nth* layer and the number of neurons in the *nth* layer, respectively. *w*_*ij*_ is the synaptic weight from the *jth* neuron in pre-synaptic layer to the *ith* neuron in the post-synaptic layer, and *o*_*j*_ ∈ {0, 1} is the neuronal output of the *jth* neuron where *o*_*j*_ = 1 denotes a spike activity and *o*_*j*_ = 0 denotes nothing occurs. Equation (4) transforms Equation (2) to an iterative update of membrane potential in the LIF model. The first item on the right refers to the decay component of neuron potential corresponding to $u\left(t_{i-1}\right)e^{\frac{t_{i-1}-t}{\tau }}$ in Equation (2), the second item *x*_*i*_ refers to the simplified representation of the pre-synaptic inputs of the *ith* neuron like $\hat{I}$(*t*) in Equation (2), and the third item *b*_*i*_ is an equivalent variable to the fire threshold. Specifically, the threshold comparison of $u_{i}^{t+1,n}=u_{i}^{t,n}f\left(o_{i}^{t,n}\right)+x_{i}^{t+1,n}+b_{i}^{n}>V_{th}$ is equivalent to $u_{i}^{t+1,n}=u_{i}^{t,n}f\left(o_{i}^{t,n}\right)+x_{i}^{t+1,n}>V_{th}-b_{i}^{n}$, hence the modeling of adjustable bias b is utilized to mimic the threshold behavior.

Actually, formulas (4)–(5) are also inspired from the LSTM model (Hochreiter and Schmidhuber, 1997; Gers et al., 1999; Chung et al., 2015) by using a forget gate *f*(.) to control the TD memory and an output gate *g*(.) to fire a spike. The forget gate *f*(.) controls the leaky extent of the potential memory in the TD, the output gate *g*(.) generates a spike activity when it is activated. Specifically, for a small positive time constant τ, *f*(.) can be approximated as:

(8) $$\begin{array}{r} f\left(o_{i}^{t,n}\right)\approx \left\{ \begin{array}{cc} \tau ,\; & o_{i}^{t,n}=0\\ 0,\; & o_{i}^{t,n}=1 \end{array}\right. \end{array}$$

since $\tau e^{-\frac{1}{\tau }}\approx 0$. In this way, the LIF model could be transformed to an iterative version where the recursive relationship in both the SD and TD is clearly describe, which is suitable for the following gradient descent training in the STD.

### 2.2. Spatio-temporal backpropagation training framework

In order to present STBP training framework, we define the following loss function *L* in which the mean square error for all samples under a given time windows *T* is to be minimized:

(9) $$\begin{array}{r} L=\frac{1}{2S}\overset{S}{\underset{s=1}{\sum }}∥y_{s}-\frac{1}{T}\overset{T}{\underset{t=1}{\sum }}{o_{s}}^{t,N}{∥}_{2}^{2} \end{array}$$

where ***y***_***s***_ and ***o***_***s***_ denote the label vector of the *sth* training sample and the neuronal output vector of the last layer *N*, respectively.

**Figure 2:**
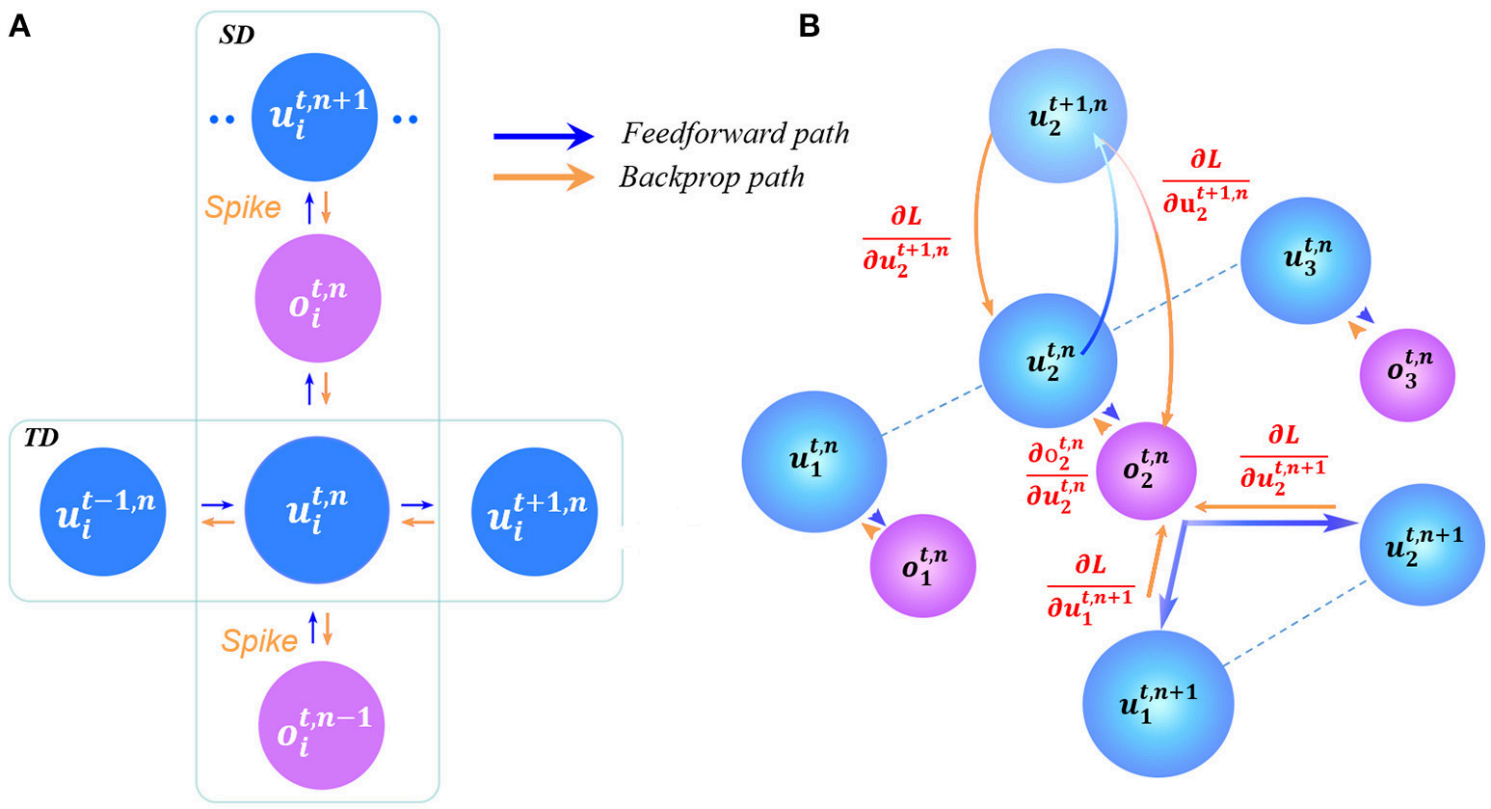
Error propagation in the STD. **(A)** At the single-neuron level, the vertical path and horizontal path represent the error propagation in the SD and TD, respectively. **(B)** Similar propagation occurs at the network level, where the error in the SD requires the multiply-accumulate operation like the feedforward computation.

By combining Equations (3)–(9) together it can be seen that *L* is a function of ***W*** and ***b***. Thus, to obtain the derivative of *L* with respect to ***W*** and ***b*** is necessary for the gradient descent. Assume that we have obtained derivative of $\frac{\partial L}{\partial o_{i}}$ and $\frac{\partial L}{\partial u_{i}}$ at each layer *n* at time *t*, which is an essential step to obtain the final $\frac{\partial L}{\partial W}$ and $\frac{\partial L}{\partial b}$. Figure 2 describes the error propagation (dependent on the derivation) in both the SD and TD at the single-neuron level (Figure 2A) and the network level (Figure 2B). At the single-neuron level, the propagation is decomposed into a vertical path of SD and a horizontal path of TD. The dataflow of error propagation in the SD is similar to the typical BP for DNNs, i.e., each neuron accumulates the weighted error signals from the upper layer and iteratively updates the parameters in different layers. While the dataflow in the TD shares the same neuronal states, which makes it quite complicated to directly obtain the analytical solution. To solve this problem, we use the proposed iterative LIF model to unfold the state space in both the SD and TD direction, thus the states in the TD at different time steps can be distinguished, which enables the chain rule for iterative propagation. Similar idea can be found in the BPTT algorithm for training RNNs in Werbos (1990).

Now, we discuss how to obtain the complete gradient descent in the following four cases. Firstly, we denote that:

(10) $$\begin{array}{rc} \delta_{i}^{t,n} & =\frac{\partial L}{\partial o_{i}^{t,n}} \end{array}$$

**Case 1:** *t* = *T* **at the output layer** *n* = *N*.

In this case, the derivative $\frac{\partial L}{\partial o_{i}^{T,N}}$ can be directly obtained since it depends on the loss function in Equation (9) of the output layer. We could have:

(11) $$\begin{array}{rc} \frac{\partial L}{\partial o_{i}^{T,N}} & =-\frac{1}{TS}\left(y_{i}-\frac{1}{T}\overset{T}{\underset{k=1}{\sum }}o_{i}^{k,N}\right). \end{array}$$

The derivation with respect to $u_{i}^{T,N}$ is generated based on $o_{i}^{T,N}$

(12) $$\begin{array}{rc} \frac{\partial L}{\partial u_{i}^{T,N}} & =\frac{\partial L}{\partial o_{i}^{T,N}}\frac{\partial o_{i}^{T,N}}{\partial u_{i}^{T,N}}=\delta_{i}^{T,N}\frac{\partial o_{i}^{T,N}}{\partial u_{i}^{T,N}}. \end{array}$$

**Case 2:** *t* = *T* **at the layers** *n* < *N*.

In this case, the derivative $\frac{\partial L}{\partial o_{i}^{T,n}}$ iteratively depends on the error propagation in the SD at time *T* as the typical BP algorithm. We have:

(13) $$\begin{array}{rc} \frac{\partial L}{\partial o_{i}^{T,n}} & =\overset{l\left(n+1\right)}{\underset{j=1}{\sum }}\delta_{j}^{T,n+1}\frac{\partial o_{j}^{T,n+1}}{\partial o_{i}^{T,n}}=\overset{l\left(n+1\right)}{\underset{j=1}{\sum }}\delta_{j}^{T,n+1}\frac{\partial g}{\partial u_{i}^{T,n}}w_{ji}. \end{array}$$

Similarly, the derivative $\frac{\partial L}{\partial u_{i}^{T,n}}$ yields

(14) $$\begin{array}{rc} \frac{\partial L}{\partial u_{i}^{T,n}} & =\frac{\partial L}{\partial o_{i}^{T,n}}\frac{\partial o_{i}^{T,n}}{\partial u_{i}^{T,n}}=\delta_{i}^{T,n}\frac{\partial g}{\partial u_{i}^{T,n}}. \end{array}$$

**Case 3:** *t* < *T* **at the output layer** *n* = *N*.

In this case, the derivative $\frac{\partial L}{\partial o_{i}^{t,N}}$ depends on the error propagation in the TD direction. With the help of the proposed iterative LIF model in Equation (3)-(5) by unfolding the state space in the TD, we acquire the required derivative based on the chain rule in the TD as follows:

(15) $$\begin{array}{l} \frac{\partial L}{\partial o_{i}^{t,N}}=\delta_{i}^{t+1,N}\frac{\partial o_{i}^{t+1,N}}{\partial o_{i}^{t,N}}+\frac{\partial L}{\partial o_{i}^{T,N}}\\ \;=\delta_{i}^{t+1,N}\frac{\partial g}{\partial u_{i}^{t+1,N}}u_{i}^{t,N}\frac{\partial f}{\partial o_{j}^{t,N}}+\frac{\partial L}{\partial o_{i}^{T,N}}, \end{array}$$

(16) $$\begin{array}{rc} \frac{\partial L}{\partial u_{i}^{t,N}} & =\frac{\partial L}{\partial u_{i}^{t+1,N}}\frac{\partial u_{i}^{t+1,N}}{\partial u_{i}^{t,N}}=\delta_{i}^{t+1,N}\frac{\partial g}{\partial u_{i}^{t+1,N}}f\left(o_{i}^{t,n}\right), \end{array}$$

where $\frac{\partial L}{\partial o_{i}^{T,N}}=-\frac{1}{TS}\left(y_{i}-\frac{1}{T}\overset{T}{\underset{k=1}{\sum }}o_{i}^{k,N}\right)$ as in Equation (11).

**Case 4:** *t* < *T* **at the layers** *n* < *N*.

In this case, the derivative $\frac{\partial L}{\partial o_{i}^{t,n}}$ depends on the error propagation in both SD and TD. On one side, each neuron accumulates the weighted error signals from the upper layer in the SD like Case 2; on the other side, each neuron also receives the propagated error from self-feedback dynamics in the TD by iteratively unfolding the state space based on the chain rule like Case 3. So we have:

(17) $$\begin{array}{rc} \frac{\partial L}{\partial o_{i}^{t,n}} & =\overset{l\left(n+1\right)}{\underset{j=1}{\sum }}\delta_{j}^{t,n+1}\frac{\partial o_{j}^{t,n+1}}{\partial o_{i}^{t,n}}+\frac{\partial L}{\partial o_{i}^{t+1,n}}\frac{\partial o_{i}^{t+1,n}}{\partial o_{i}^{t,n}} \end{array}$$

(18) $$\begin{array}{r} =\overset{l\left(n+1\right)}{\underset{j=1}{\sum }}\delta_{j}^{t,n+1}\frac{\partial g}{\partial u_{i}^{t,n}}w_{ji}+\delta_{i}^{t+1,n}\frac{\partial g}{\partial u_{i}^{t,n}}u_{i}^{t,n}\frac{\partial f}{\partial o_{i}^{t,n}}, \end{array}$$

(19) $$\begin{array}{rc} \frac{\partial L}{\partial u_{i}^{t,n}} & =\frac{\partial L}{\partial o_{i}^{t,n}}\frac{\partial o_{i}^{t,n}}{\partial u_{i}^{t,n}}+\frac{\partial L}{\partial o_{i}^{t+1,n}}\frac{\partial o_{i}^{t+1,n}}{\partial u_{i}^{t,n}} \end{array}$$

(20) $$\begin{array}{r} =\delta_{i}^{t,n}\frac{\partial g}{\partial u_{i}^{t,n}}+\delta_{i}^{t+1,n}\frac{\partial g}{\partial u_{i}^{t+1,n}}f\left(o_{i}^{t,n}\right). \end{array}$$

Based on the four cases, the error propagation procedure (depending on the above derivatives) is shown in Figure 2. At the single-neuron level (Figure 2A), the propagation is decomposed into the vertical path of SD and the horizontal path of TD. At the network level (Figure 2B), the dataflow of error propagation in the SD is similar to the typical BP for DNNs, i.e. each neuron accumulates the weighted error signals from the upper layer and iteratively updates the parameters in different layers; and in the TD, the neuronal states are iteratively unfolded in the timing dimension that enables the chain-rule propagation. Finally, we obtain the derivatives with respect to ***W*** and ***b*** as follows:

(21) $$\begin{array}{rc} \frac{\partial L}{\partial b^{n}} & =\overset{T}{\underset{t=1}{\sum }}\frac{\partial L}{\partial u^{t,n}}\frac{\partial u^{t,n}}{\partial Lb^{n}}=\overset{T}{\underset{t=1}{\sum }}\frac{\partial L}{\partial u^{t,n}}, \end{array}$$

(22) $$\begin{array}{l} \frac{\partial L}{\partial W^{n}}=\overset{T}{\underset{t=1}{\sum }}\frac{\partial L}{\partial u^{t,n}}\frac{\partial u^{t,n}}{\partial W^{n}}\\ \;=\overset{T}{\underset{t=1}{\sum }}\frac{\partial L}{\partial u^{t,n}}\frac{\partial u^{t,n}}{\partial x^{t,n}}\frac{\partial x^{t,n}}{\partial W^{n}}=\overset{T}{\underset{t=1}{\sum }}\frac{\partial L}{\partial u^{t,n}}{o^{t,n-1}}^{T}, \end{array}$$

where $\frac{\partial L}{\partial u^{t,n}}$ can be obtained from in Equation (11)–(17). Given the ***W*** and ***b*** according to the STBP, we can use gradient descent optimization algorithms to effectively train SNNs for achieving high performance.

### 2.3. Derivative approximation of the non-differentiable spike activity

**Figure 3:**
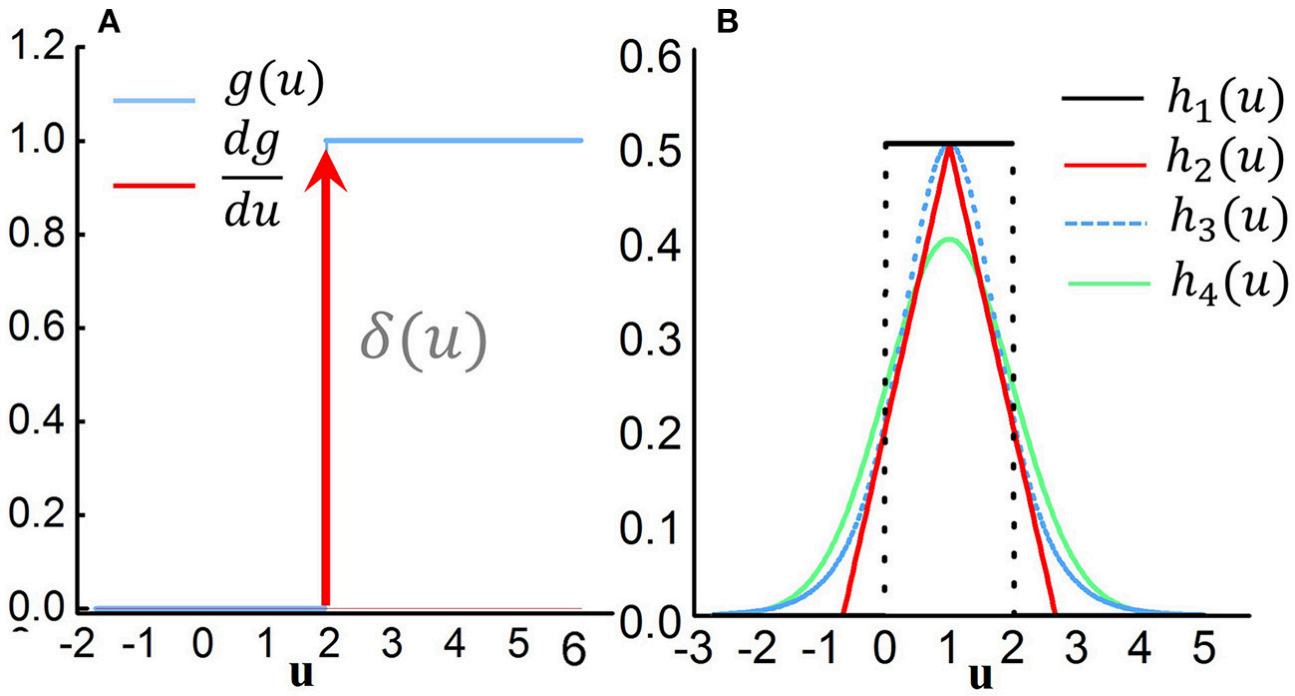
Derivative approximation of the non-differentiable spike activity. **(A)** Step activation function of the spike activity and its original derivative function which is a typical Diract function δ(*u*) with infinite value at *u* = 0 and zero value at other points. This non-differentiable property disables the error propagation. **(B)** Several typical curves to approximate the derivative of spike activity.

In the previous sections, we have presented how to obtain the gradient information based on STBP, but the issue of non-differentiable points at each spiking time is yet to be addressed. Actually, the derivative of output gate *g*(*u*) is required for the STBP training of Equation (11)–(21). Theoretically, *g*(*u*) is a non-differentiable Dirac function of δ(*u*) which greatly challenges the effective learning of SNNs (Lee et al., 2016). *g*(*u*) has zero value everywhere except an infinity value at zero, which causes the gradient vanishing or exploding issue that disables the error propagation. One of existing method views the discontinuous points of the potential at spiking times as noise and claimed it is beneficial for the model robustness (Bengio et al., 2015; Lee et al., 2016), while it did not directly address the non-differentiability of the spike activity. To this end, we introduce four curves to approximate the derivative of spike activity denoted by *h*_1_, *h*_2_, *h*_3_, and *h*_4_ in Figure 3B:

(23) $$\begin{array}{rc} h_{1}\left(u\right) & =\frac{1}{a_{1}}sign\left(|u-V_{th}|<\frac{a_{1}}{2}\right), \end{array}$$

(24) $$\begin{array}{rc} h_{2}\left(u\right) & =\left(\frac{\sqrt{a_{2}}}{2}-\frac{a_{2}}{4}|u-V_{th}|\right)sign\left(\frac{2}{\sqrt{a_{2}}}-|u-V_{th}|\right), \end{array}$$

(25) $$\begin{array}{rc} h_{3}\left(u\right) & =\frac{1}{a_{3}}\frac{e^{\frac{V_{th}-u}{a_{3}}}}{{\left(1+e^{\frac{V_{th}-u}{a_{3}}}\right)}^{2}}, \end{array}$$

(26) $$\begin{array}{rc} h_{4}\left(u\right) & =\frac{1}{\sqrt{2\pi a_{4}}}e^{-\frac{{\left(u-V_{th}\right)}^{2}}{2a_{4}}}, \end{array}$$

where *a*_*i*_(*i* = 1, 2, 3, 4) determines the curve steepness, i.e., the peak width. In fact, *h*_1_, *h*_2_, *h*_3_, and *h*_4_ are the derivative of the rectangular function, polynomial function, sigmoid function and Gaussian cumulative distribution function, respectively. To be consistent with the Dirac function δ(*u*), we introduce the coefficient *a*_*i*_ to ensure the integral of each function is 1. Obviously, it can be proven that all the above candidates satisfy that:

(27) $$\begin{array}{r} \underset{a_{i}\to 0^{+}}{\lim}h_{i}\left(u\right)=\frac{dg}{du},i=1,2,3,4. \end{array}$$

Thus, $\frac{\partial g}{\partial u}$ in Equation (11)–(21) for STBP can be approximated by:

(28) $$\begin{array}{r} \frac{\partial g}{\partial u}\approx h_{i}\left(u\right),i=1,2,3,4. \end{array}$$

In section 3.3, we will analyze the influence on the SNNs performance with different curves and different values of *a*_*i*_.

## 3. Results

### 3.1. Parameter initialization

The initialization of parameters, such as the weights, thresholds and other parameters, is crucial for stabilizing the firing activities of the whole network. We should simultaneously ensure timely response of pre-synaptic stimulus but avoid too much spikes that reduces the neuronal selectivity. As it is known that the multiply-accumulate operations of the pre-spikes and weights, and the threshold comparison are two key computation steps in the forward pass. This indicates the relative magnitude between the weights and thresholds determines the effectiveness of parameter initialization. In this paper, we fix the threshold to be constant in each neuron for simplification, and only adjust the weights to control the activity balance. Firstly, we initial all the weight parameters by sampling from the standard uniform distribution:

(29) $$\begin{array}{r} W\sim U\left[-1,1\right] \end{array}$$

Then, we normalize these parameters by:

(30) $$\begin{array}{rc} w_{ij}^{n} & =\frac{w_{ij}^{n}}{\sqrt{\sum_{j=1}^{l\left(n-1\right)}{w_{ij}^{n}}^{2}}},\;i=1,..,l\left(n\right) \end{array}$$

The set of other parameters is presented in Table 1. Note that Adam (adaptive moment estimation Kingma and Ba, 2014) is a popular optimization method to accelerate the convergence speed of the gradient descent. When updating the parameters (W and b) based on their gradients (in Equation 21-22), we apply Adam optimizer that is usually used in ANNs. Actually, it does not affect the process of gradient acquire process, while just used for parameter update. The corresponding parameters are also listed in Table 1. Furthermore, throughout all the simulations in our work, any complex skill as in Diehl et al. (2015); Lee et al. (2016) is no longer required, such as the error normalization, weight/threshold regularization, fixed-amount-proportional reset mechanism, etc.

**Table 1:** Parameters set in our experiments.

**Network parameter**	**Description**	**Value**
*T*	Time window	30 ms
*V*_*th*_	Threshold (MNIST/object detection dataset/N-MNIST)	1.5, 2.0, 0.2
τ	Decay factor (MNIST/object detection dataset/N-MNIST)	0.1, 0.15, 0.2 ms
*a*_1_, *a*_2_, *a*_3_, *a*_4_	Derivative approximation parameters (Figure 3)	1.0
*dt*	Simulation time step	1 ms
*r*	Learning rate (SGD)	0.5
β_1_, β_2_, λ	Adam parameters	0.9, 0.999, 1-10^−8^

### 3.2. Dataset experiments

We test the STBP training framework on various datasets, including the static MNIST dataset, a custom object detection dataset as well as the dynamic N-MNIST dataset.

#### 3.2.1. Spatio-temporal fully connected neural network

##### 3.2.1.1. Static dataset

**Figure 4:**
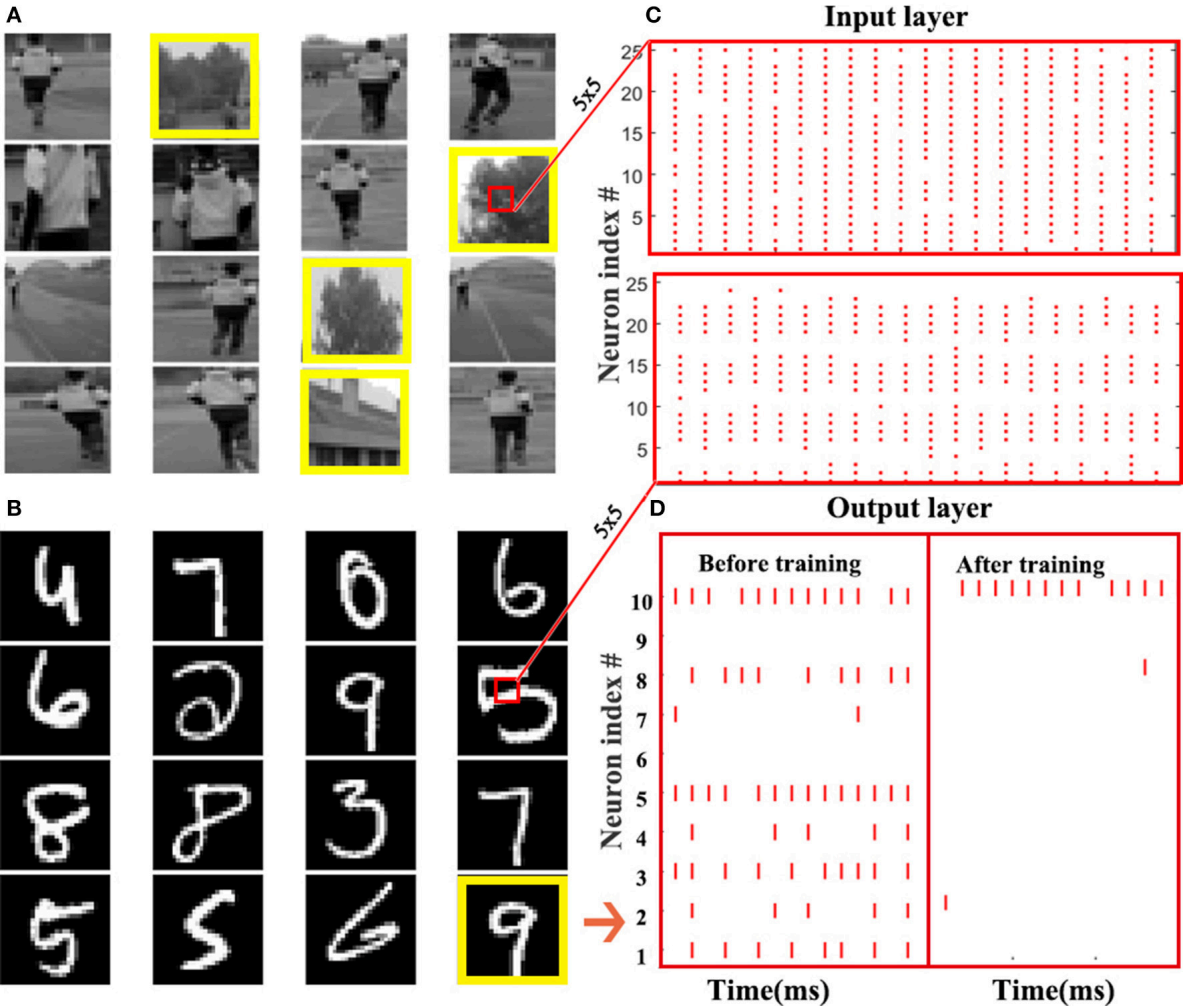
Static dataset experiments. **(A)** A custom dataset for object detection. This dataset is a two-category image set built by our lab for pedestrian detection. By detecting whether there is a pedestrian, an image sample is labeled by 0 or 1. The images in the yellow boxes are labeled as 1, and the rest ones are marked as 0. **(B)** MNIST dataset. **(C)** Raster plot of the spike pattern converted from the center patch of 5 × 5 pixels of a sample in the object detection dataset (up) and MNIST (down). **(D)** Raster plot presents the comparison of output spike pattern over a digit 9 in MNIST dataset before and after the STBP training.

The MNIST dataset of handwritten digits (Lecun et al., 1998) (Figure 4B) and a custom dataset for object detection (Zhang et al., 2016) (Figure 4A) are chosen to test our method. MNIST is comprised of a training set with 60,000 labeled hand-written digits, and a testing set of other 10,000 labeled digits, which are generated from the postal codes of 0-9. Each digit sample is a 28 × 28 grayscale image. The object detection dataset is a two-category image dataset created by our lab for pedestrian detection. It includes 1,509 training samples and 631 testing samples of 28 × 28 grayscale image. Actually, these images are patches in many real-world large-scale pictures, where each patch corresponds to an intrinsic location. The patches are sent to a neural network for binary classification to tell us whether or not an object exists in the scene, which is labeled by 0 or 1, as illustrated in Figure 4A. If so, an extra model will use the intrinsic location of this patch as the detected location. The input of the first layer should be a spike train, which requires us to convert the samples from the static datasets into spike events. To this end, the Bernoulli sampling conversion from original pixel intensity to the spike rate is used in this paper. Specifically, each normalized pixel is probabilistically converted to a spike event (“1”) at each time step by using an independent and identically distributed Bernoulli sampling. The probability of generating a “1,” i.e., a spike event, is proportional to the normalized value of the entry. For example, if the pixel intensity is 0.8, it can generate a spike event at each time step with probability of 0.8 ant remain silent (“1”) with probability of 0.2 = 1 − 0.8. Then, the spike events within a certain time window form a spike train. The upper and lower sub-figures in Figure 4C are the spike pattern of 25 input neurons converted from the center patch of 5 × 5 pixels of a sample on the object detection dataset and MNIST, respectively. Figure 4D illustrates an example for the spike pattern of output layer within 15ms before and after the STBP training over the stimulus of digit 9. At the beginning, neurons in the output layer randomly fires, while after the training the 10*th* neuron coding digit 9 fires most intensively that indicates correct inference is achieved.

Table 2 compares our method with several other advanced results that uses the structure similar to Multi-layer Perceptron (MLP). Although we do not use any complex skill, the proposed STBP training method also outperforms all the reported results. We can achieve 98.89% testing accuracy which performs the best. Table 3 compares our model with the typical MLP on the object detection dataset. The baseline model is one of the typical artificial neural networks (ANNs), i.e., not SNNs, and in the following we use ‘non-spiking network’ to distinguish them. It can be seen that our model achieves comparable performance with the non-spiking MLP. Note that the overall firing rate of the input spike train from the object detection dataset is higher than the one from MNIST dataset, so we increase its threshold to 2.0 in the simulation experiments.

**Table 2:** Comparison with the state-of-the-art spiking networks with similar architecture on MNIST.

**Model**	**Network structure**	**Training skills**	**Accuracy%**
Spiking RBM (STDP) (Neftci et al., 2013)	784-500-40	None	93.16
Spiking RBM(pre-training^*^) (Peter et al., 2013)	784-500-500-10	None	97.48
Spiking MLP(pre-training^*^) (Diehl et al., 2015)	784-1200-1200-10	Weight normalization	98.64
Spiking MLP(pre-training^*^) (Hunsberger and Eliasmith, 2015)	784-500-200-10	None	98.37
Spiking MLP(BP) (O'Connor and Welling, 2016)	784-200-200-10	None	97.66
Spiking MLP(STDP) (Diehl and Cook, 2015)	784-6400	None	95.00
Spiking MLP(BP) (Lee et al., 2016)	784-800-10	Error normalization/ parameter regularization	98.71
Spiking MLP(STBP)	784-800-10	None	**98.89**

We mainly compare with these methods that have the similar network architecture, and

* *means that their model is based on pre-trained ANN models*.

**Table 3:** Comparison with the typical MLP over object detection dataset.

**Model**	**Network structure**	**Accuracy**	
		**Mean**	Interval^*^
Non-spiking MLP(BP)	784-400-10	98.31%	[97.62%, 98.57%]
Spiking MLP(STBP)	784-400-10	**98.34%**	[**97.94%**, **98.57%**]

* Results with epochs [201,210].

##### 3.2.1.2. Dynamic dataset

**Figure 5:**
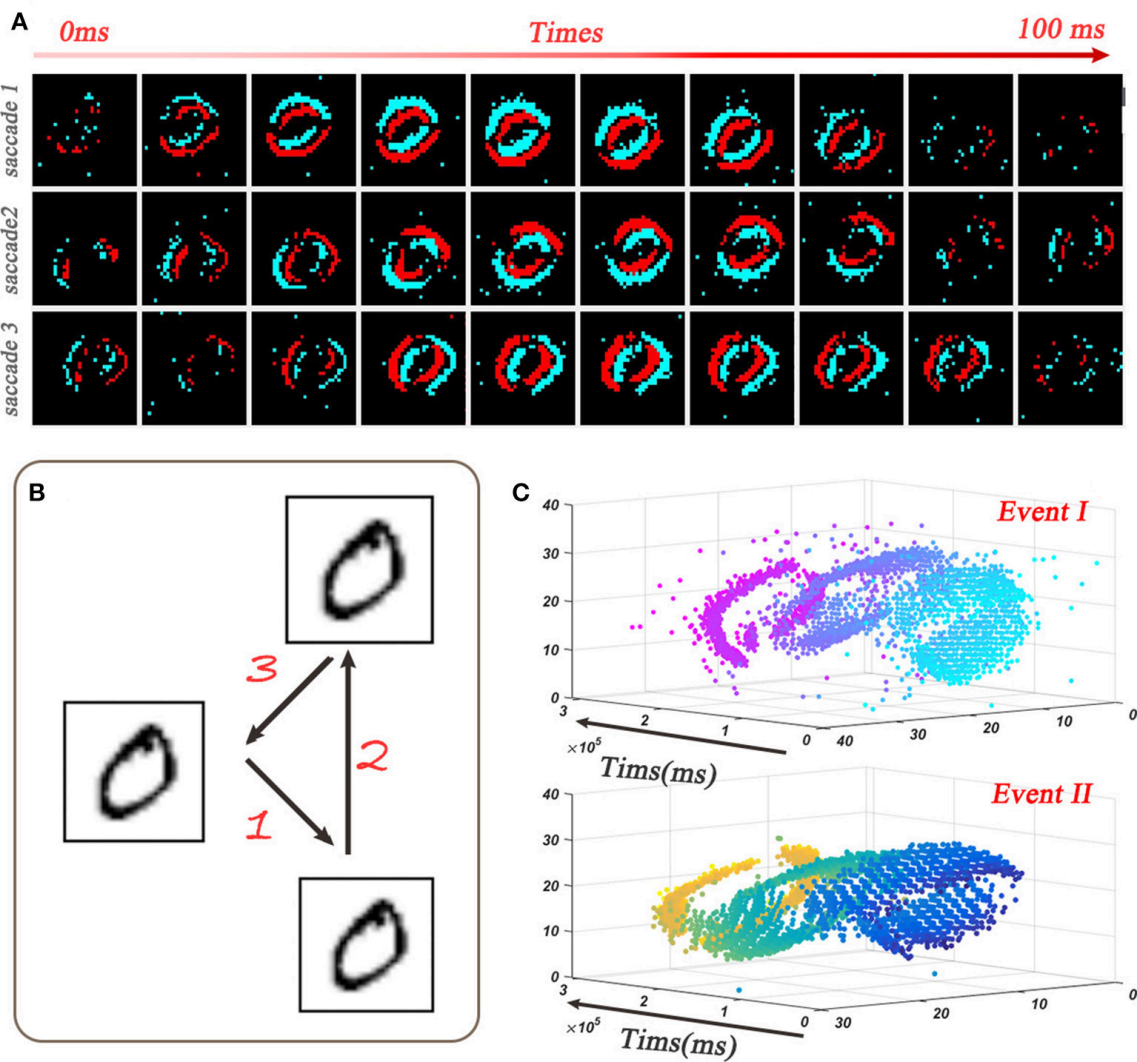
Dynamic dataset of N-MNIST. **(A)** Each sub-picture shows a 10ms-width spike train during the saccades. **(B)** Spike train is generated by moving the dynamic vision sensor (DVS) in turn toward the direction of 1, 2, and 3. **(C)** Spatio-temporal representation of the spike train from digit 0 (Orchard et al., 2015)where the upper one and lower one denote the on-events and off-events, respectively.

Compared with the static dataset, dynamic dataset, such as the N-MNIST (Orchard et al., 2015), contains richer temporal features, and therefore it is more suitable to exploit SNN's potential ability. We use the N-MNIST database as an example to evaluate the capability of our STBP method on dynamic dataset. N-MNIST converts the mentioned static MNIST dataset into its dynamic version of spike train by using the dynamic vision sensor (DVS) (Lichtsteiner et al., 2007). For each original sample from MNIST, the work (Orchard et al., 2015) controls the DVS to move in the direction of three sides of the isosceles triangle in turn (Figure 5B) and collects the generated spike train which is triggered by the intensity change at each pixel. Figure 5A records the saccade results on digit 0. Each sub-graph records the spike train within 10ms and each 100ms represents one saccade period. Due to the two possible change directions of each pixel intensity (brighter or darker), DVS could capture the corresponding two kinds of spike events, denoted by on event and off event, respectively (Figure 5C). Since N-MNIST allows the relative shift of images during the saccade process, it produces 34 × 34 pixel range. And from the spatio-temporal representation in Figure 5C, we can see that the on-events and off-events are so different that we use two channel to distinguish it. Therefore, the network structure is 34 × 34 × 2-400-400-10.

Table 4 compares our STBP method with some state-of-the-art results on N-MNIST dataset. The upper 5 results are based on ANNs, and lower 4 results including our method uses SNNs. The ANNs methods usually adopt a frame-based method, which collects the spike events in a time interval (30 ~ 300*ms*) to form a frame of image, and use the conventional algorithms for image classification to train the networks. Since the transformed images are often blurred, the frame-based preprocessing is harmful for model performance and abandons the hardware friendly event-driven paradigm. As can be seen from Table 4, the models of ANN are generally worsen than the models of SNNs.

**Table 4:** Comparison with state-of-the-art networks over N-MNIST.

**Model**	**Network structure**	**Training skills**	**Accuracy%**
Non-spiking CNN(BP) (Neil et al., 2016)	-	None	95.30
Non-spiking CNN(BP) (Neil and Liu, 2016)	-	None	98.30
Non-spiking MLP(BP)(Lee et al., 2016)	34 × 34 × 2-800-10	None	97.80
LSTM(BPTT) (Neil et al., 2016)	-	Batch normalization	97.05
Phased-LSTM(BPTT) (Neil et al., 2016)	-	None	97.38
Spiking CNN(pre-training^*^) (Neil and Liu, 2016)	-	None	95.72
Spiking MLP(BP) (Lee et al., 2016)	34 × 34 × 2-800-10	Error normalization/parameter regularization	98.74
Spiking MLP(BP) (Cohen et al., 2016)	34 × 34 × 2-10000-10	None	92.87
Spiking MLP(STBP)	34 × 34 × 2-800-10	None	**98.78**

We only show the network structure based on MLP, and the other network structure refers to the above references.

* *means that their model is based on pre-trained ANN models*.

In contrast, SNNs could naturally handle event stream patterns, and via better use of spatio-temporal features, our proposed STBP method achieves best accuracy of 98.78% when compared all the reported ANNs and SNNs methods. The greatest advantage of our method is that we did not use any complex training skill, which is beneficial for future hardware implementation.

#### 3.2.2. Spatio-temporal convolution neural network

Extending our framework to convolution neural network structure allows the network going deeper and grants network more powerful SD information. Here we use our framework to establish the spatio-temporal convolution neural network. Compared with our spatio-temporal fully connected network, the main difference is the processing of the input image, where we use the convolution in place of the weighted summation. Specifically, in the convolution layer, each convolution neuron receives the convoluted results as input and updates its state according to the LIF model. In the pooling layer, because the binary coding of SNNs is inappropriate for standard max pooling, we use the average pooling instead.

Our spiking CNN model are tested on the MNIST dataset as well as the object detection dataset. In the MNIST, our network contains two convolution layers with kernel size of 5 × 5 and two average pooling layers alternatively, followed by one full connected layer. And like traditional CNN, we use the elastic distortion (Simard et al., 2003) to preprocess dataset. Table 5 records the state-of-the-art performance of spiking convolution neural networks over MNIST dataset. Our proposed spiking CNN model obtain 98.42% accuracy, which outperforms other reported spiking networks with slightly lighter structure. Furthermore, we configure the same network structure on a custom object detection database to evaluate the proposed model performance. The testing accuracy is reported after training 200 epochs. Table 6 indicates our spiking CNN model could achieve a competitive performance with the non-spiking CNN.

**Table 5:** Comparison with other spiking CNN over MNIST.

**Model**	**Network structure**	**Accuracy**
Spiking CNN (pre-training^*^) (Esser et al., 2016)	28 × 28 × 1-12C5-P2-64C5-P2-10	99.12%
Spiking CNN(BP) (Lee et al., 2016)	28 × 28 × 1-20C5-P2-50C5-P2-200-10	99.31%
Spiking CNN (STBP)	28 × 28 × 1-15C5-P2-40C5-P2-300-10	**99.42%**

We mainly compare with these methods that have the similar network architecture, and

* means that their model is based on pre-trained ANN models.

**Table 6:** Comparison with the typical CNN over object detection dataset.

**Model**	**Network structure**	**Accuracy**	
		**Mean**	Interval^*^
Non-spiking CNN(BP)	28 × 28 × 1-6C3-300-10	98.57%	[98.57%, 98.57%]
Spiking CNN(STBP)	28 × 28 × 1-6C3-300-10	**98.59%**	[**98.26%**, **98.89%**]

* *Results with epochs [201,210]*.

### 3.3. Performance analysis

#### 3.3.1. The impact of derivative approximation curves

**Figure 6:**
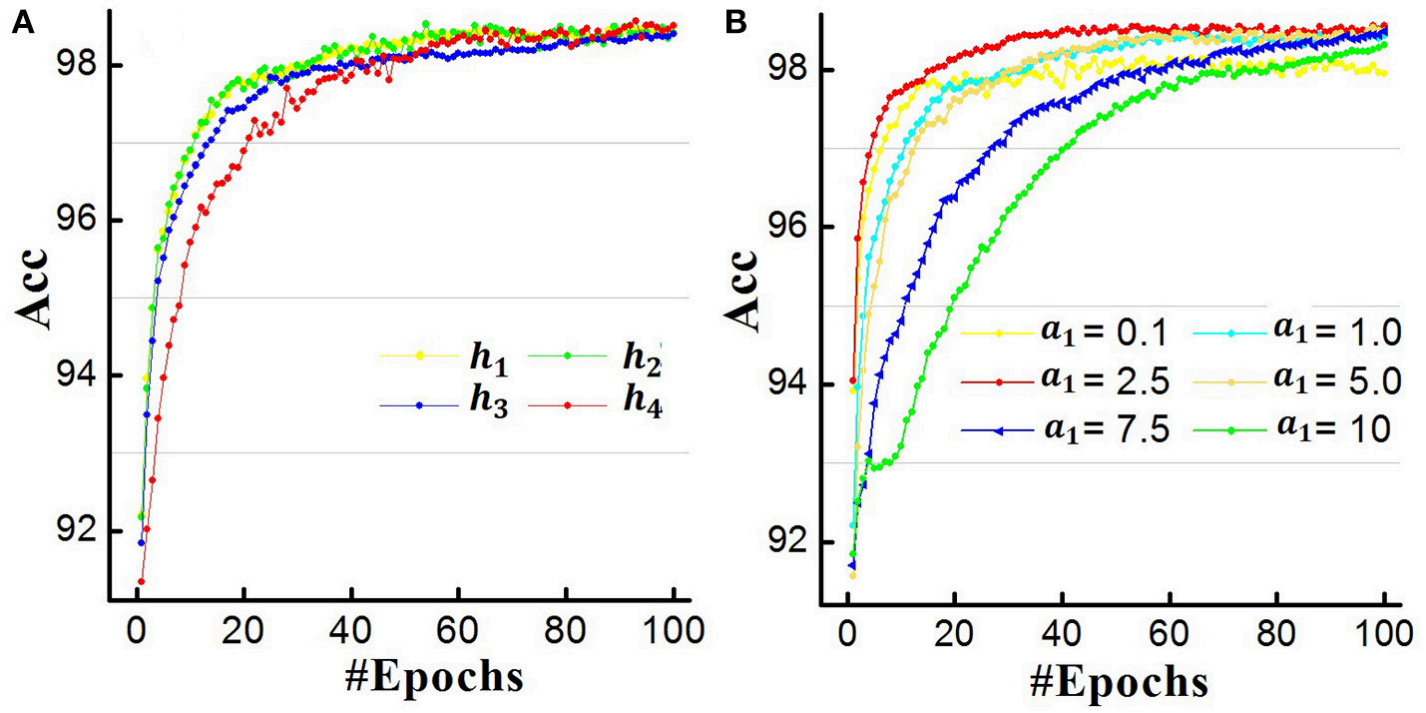
Comparisons of different derivation approximation curves. **(A)** The influence of curve shape. **(B)** The influence of curve steepness/width.

In subsection 2.3, we introduce different curves to approximate the ideal derivative of the spike activity. Here we try to analyze the influence of different approximation curves on the testing accuracy. The experiments are conducted on the MNIST dataset, and the network structure is 784−400−10. The testing accuracy is reported after training 200 epochs. Firstly, we compare the impact of different curve shapes on model performance. In our simulation we use the mentioned *h*_1_, *h*_2_, *h*_3_, and *h*_4_ shown in Figure 3B. Figure 6A illustrates the results of approximations of different shapes. We observe that different nonlinear curves, such as *h*_1_, *h*_2_, *h*_3_, and *h*_4_, only present small variations on the performance.

Furthermore, we use the rectangular approximation as an example to explore the impact of curve steepness (or peck width) on the experiment results. We set *a*_1_ = 0.1, 1.0, 2.5, 5.0, 7.5, 10 and the corresponding results are plotted in Figure 6B. Different colors denote different *a*_1_ values. Actually, *a*_1_ in the rang of 0.5–5.0 achieves comparable convergence while too large (*a*_1_ = 10) or too small (*a*_1_ = 0.1) value performs worse performance. Combining Figures 6A,B, it indicates that the key point for approximating the derivation of the spike activity is to capture the nonlinear nature and proper curve steepness, while the specific curve shape is not so critical.

#### 3.3.2. The impact of temporal domain

A major contribution of this work is introducing the temporal domain into the existing spatial domain based BP training method, which makes full use of the spatio-temporal dynamics of SNNs and enables the high-performance training. Now we quantitatively analyze the impact of the TD item. The experiment configurations keep the same with the previous section (784 − 400 − 10) and we also report the testing results after training 200 epochs. Here the existing BP in the SD is termed as SDBP.

Table 7 records the simulation results. The testing accuracy of SDBP is lower than the accuracy of the STBP on different datasets, which shows the temporal information is beneficial for model performance. Specifically, compared to the STBP, the SDBP has a 1.21% loss of accuracy on the objective recognition dataset, which is 5 times larger than the loss on the MNIST. And results also imply that the performance of SDBP is not stable enough. In addition to the interference of the dataset itself, the reason for this variation may be the unstability of SNNs training method. Actually, the training of SNNs relies heavily on the parameter initialization, which is also a great challenge for SNNs applications. In many reported works, researchers usually leverage some special skills or mechanisms to improve the training performance, such as the regularization, normalization, etc. In contrast, by using our STBP training method, much higher performance can be achieved on the same network (98.48% on MNIST and 98.32% on the object detection dataset). Note that the STBP didn't use any complex training skill. This stability and robustness indicate that the dynamics in the TD fundamentally includes great potential for the SNNs computing and this work indeed provides an insightful evidence.

**Table 7:** Comparison for the SDBP model and the STBP model on different datasets.

**Model**	**Dataset**	**Network structure**	**Training skills**	**Accuracy**	
				**Mean**	Interval^*^
Spiking MLP	Objective recognition	784-400-10	None	97.11%	[96.04%,97.78%]
(SDBP)	MNIST	784-400-10	None	98.29%	[98.23%, 98.39%]
Spiking MLP	Objective recognition	784-400-10	None	**98.32%**	[**97.94%**, **98.57%**]
(STBP)	MNIST	784-400-10	None	**98.48%**	[**98.42%**, **98.51%**]

* *Results with epochs [201,210]*.

## 4. Discussion

In this work, we propose a spatio-temporal backpropagation (STBP) algorithm that allows to effective supervised learning for SNNs. Although existing supervised learning methods have considered either SD feature or TD feature (Gtig and Sompolinsky, 2006; Lee et al., 2016; O'Connor and Welling, 2016), they do not combine them well, which may cause their model hardly to get high-accuracy results on some standard benchmarks. Although indirect training methods (Diehl et al., 2015; Hunsberger and Eliasmith, 2015) achieve performance very close to the pre-trained model, the conversion strategy essentially helps little to understand the nature of SNNs. By combining the information in both SD and TD domain, our STBP algorithm can bridge this gap. We implement STBP on both MLP and CNN architecture, which are verified on both static and dynamic datasets. Results of our model are superior to the existing state-of-the-art SNNs on relatively small-scale networks of spiking MLP and CNNs, and even outperforms non-spiking DNNs with the same network size on dynamic N-MNIST dataset. Specifically, on MNIST, we achieve 98.89% accuracy with fully connected architecture and achieve 99.42% accuracy with convolutional architecture. On N-MNIST, we achieve 98.78% accuracy with fully connected architecture, to the best of our knowledge, which beats previous works on this dataset.

Furthermore, we introduce an approximated derivative to address the non-differentiable issue of the spike activity. Previous works regard the non-differentiable points as noise (Vincent et al., 2008; Hunsberger and Eliasmith, 2015), while our results reveal that the steepness and width of the approximation curve would affect the learning performance, while the specific curve shape is not so critical. Another attractive advantage of our algorithm is that it does not need complex training skills which are widely used in existing schemes to guarantee the performance (Diehl et al., 2015; Lee et al., 2016; Neil et al., 2016), that makes it easier to be implemented in large-scale networks. These results also indicate that the use of spatio-temporal complexity to solve problems captures one of the key potentials of SNNs. Because the brain leverages complexity in both the temporal and spatial domain to solve problems, we also would like to claim that implementing the STBP on SNNs is more bio-plausible than applying the spatial BP like that in DNNs. The remove of extra training skills also makes it more hardware-friendly for the design of neuromorphic chips with online learning ability.

Since the N-MNIST converts the static MNIST into a dynamic event-driven version by the relative movement of DVS, in essence this generation method could not provide sufficient temporal information and additional data feature than original database. Hence it is important to further apply our model to tackle more convincing problems with temporal characteristics, such as TIMIT (Garofolo et al., 1993), Spoken Digits database (Davis et al., 1952).

We also evaluate our model on CIFAR-10 dataset. Here we do not resort to any data argument methods and training techniques (e.g., batch normalization, weight decay). Considering the training speed, we adopt a small-scale structure with 2 convolution layers (20 channels with kernel 5 × 5 - 30 channels 5 × 5), 2 × 2 average-pooling layers after each convolution layer, followed by 2 fully connected layers (256 and 10 neurons, respectively). Testing accuracy is reported after 100 training epochs. The spiking CNN achieves 50.7% accuracy and the ANN with same structure achieves 52.9% accuracy. It suggests that SNN is able to obtain comparable performance on larger datasets. To the best of our knowledge, currently few works report the results on CIFAR10 for direct training of SNNs (not including those pre-trained ANN models). The difficulty of this problem mainly involves two aspects. Firstly, it is challenging to implement BP algorithm to train SNNs directly at this stage because of the complex dynamics and non-differentiable spike activity. Secondly, although it is energy efficient to realize SNN on specialized neuromorphic chips, it is very difficult and time-consuming to simulate the complex kinetic behaviors of SNN on computer software (about ten times or even hundred times the runtimes compared to the same structure ANN). Therefore, accelerating the supervised training of large scale SNNs based on CPU/GPU or neuromorphic substrates is also worth studying in the future.

## Author contributions

YW and LD proposed the idea, designed and did the experiments. YW, LD, GL, and JZ conducted the modeling work. YW, LD, and GL wrote the manuscript, then JZ and LS revised it. LS directed the projects and provided overall guidance.

### Conflict of interest statement

The authors declare that the research was conducted in the absence of any commercial or financial relationships that could be construed as a potential conflict of interest.
